# Temporomandibular joint disc position affects condylar bone modeling – a randomized controlled clinical trial

**DOI:** 10.1097/JS9.0000000000002926

**Published:** 2025-07-17

**Authors:** Shijing Yue, Jiong Zhao, Siyu Wu, Qianyang Xie, Yi Luo, Xin Nie, Minjie Chen, Guo Bai, Chi Yang

**Affiliations:** a Department of Oral Surgery, Shanghai Ninth People’s Hospital, Shanghai Jiao Tong University School of Medicine; College of Stomatology, Shanghai Jiao Tong University; National Center for Stomatology; National Clinical Research Center for Oral Diseases; Shanghai Key Laboratory of Stomatology; Shanghai Research Institute of Stomatology; Research Unit of Oral and Maxillofacial Regenerative Medicine, Chinese Academy of Medical Sciences, Shanghai, China; b Biostatistics Office of Clinical Research Unit, Shanghai Ninth People’s Hospital, Shanghai Jiao Tong University School of Medicine, Shanghai, China

**Keywords:** anterior disc displacement of temporomandibular joint, condylar bone modeling and remodeling, disc repositioning surgery, temporomandibular disc position

## Abstract

**Backgrounds:**

The primary controversy surrounding the treatment of anterior disc displacement (ADD) of temporomandibular joints (TMJ) stems from the unclear relationship between disc position and condylar adaptation. The long-term impact of varying disc position on condylar bone remodeling remains poorly understood.

**Methods:**

A randomized controlled clinical trial was conducted to evaluate the effects of different disc positions on condylar bone modeling/remodeling at baseline (T0) and after an 18-month follow-up (T1). Patients under 25 years of age diagnosed with TMJ ADD without reduction (ADDwoR) were enrolled and randomly allocated in a 1:1 ratio to either the disc displaced (DD) group or the disc reposited (DR) group. Participants in the DR group underwent arthroscopic disc repositioning surgery, whereas those in the DD group received no occlusal or surgical intervention. CT scans were obtained at T0 and T1, and three-dimensional condylar reconstructions were generated using Mimics 21.0 software. Seven anatomical points were defined on the condylar surface to represent distinct regions. The condylar volume and vertical height of 7 points were measured for the analysis.

**Results:**

This study included 60 patients with ADDwoR. At T1, the DR group demonstrated a significant increase in total condylar volume of 279.6 mm^3^ per patient, compared to a slight 29.5 mm^3^ increase in the DD group (*P* < 0.0001). Differential height changes were observed between groups, particularly at the posterolateral and lateral condylar surfaces (*P* < 0.05). Besides, anterolaterally displaced discs were associated with bone resorption at the posteromedial condyle, in contrast to anteromedially displaced discs at T0 (*P* < 0.01).

**Conclusions:**

This study provides compelling evidence that ADDwoR restricts condylar bone growth in adolescents and young adults. Furthermore, disc position directly determines patterns of condylar bone modeling and remodeling in this population.

## Introduction

This work is compliant with the TITAN Guidelines 2025 for transparent artificial intelligence (AI) use[1]. The authors utilized DeepSeek-V3 for language enhancement during manuscript preparation. After using this tool, the authors reviewed and edited the content as needed and take full responsibility for the content.

Temporomandibular joints serve as the crucial articulations between the mandible and the skull, facilitating essential functions in mastication, speech, and mandibular movement[2]. The TMJ articular disc is positioned superior to the mandibular condyle at the resting position[2]. Anterior displacement of the disc from its physiological position results in TMJ anterior disc displacement (ADD), which is the most common type of TMJ disease. ADD predominantly affects adolescents and young women with symptoms such as clicking, joint pain, and restricted mandibular mobility^[3,4]^.

Condyle plays a pivotal role in mandibular growth, especially during adolescence. Condylar bone resorption, as a degenerative bone remodeling process, compromises mandibular development and leads to maxillofacial abnormalities, including mandibular deviation and retrusion^[5-12]^. Therefore, condylar bone assessment is crucial for predicting mandibular growth and facial asymmetry. The position of the articular disc shows a strong correlation with condylar bone modeling and remodeling patterns. Persistent disc displacement has been associated with condylar resorption, as evidenced by longitudinal studies demonstrating decreased condylar height in ADD patients over 6 months of follow-up, as well as condylar volume reduction correlating with disc displacement deteriorates^[13,14]^. In contrast, both condylar volume and height demonstrate significant improvement following disc repositioning surgery^[13,15]^. Notably, emerging evidence suggests that postsurgical bone formation occurs asymmetrically across the condylar surface, with 53.5% of new bone deposition localized to the posterior slope at 1-year follow-up^[16,17]^. These findings collectively support the hypothesis that disc position significantly influences condylar bone modeling and remodeling.

However, how disc position influences condylar bone modeling and remodeling remains unclear. Current literature lacks robust long-term prospective cohort studies on their relationship. Methodologically, most existing studies have assessed the condylar bone changes using single MRI slices, which cannot provide a comprehensive three-dimensional evaluation of condylar bone modeling and remodeling. To address these limitations, a randomized clinical trial was conducted to systematically evaluate how disc position affects condylar bone modeling/remodeling in young patients for 18 months using CT-based analysis. This study established a novel, comprehensive imaging evaluation protocol for condylar bone assessment. Young patients with anterior disc displacement without reduction (ADDwoR) were enrolled and randomly allocated to either the disc displaced (DD) group or the disc reposited (DR) group. The DR group underwent disc repositioning surgery via an arthroscopic approach, while the DD group received no occlusal or surgical intervention. All participants underwent head CT scans at baseline and 18 months later (18 months post-surgery for the DR group). Three-dimensional condylar reconstructions were generated using specialized software, enabling precise quantification of both condylar volume and height as key indicators of bone modeling and remodeling process.

## Methods and patients

### Study design

This was an investigator-initiated, open-label, randomized, controlled clinical trial and was approved by Shanghai Ninth Hospital. Participants and their legal guardians received both written and verbal information about the trial and signed consent forms. This work has been reported in line with the Consolidated Standards of Reporting Trials (CONSORT) criteria[18].

### Patients

Patients with TMJ ADDwoR were included in this study from 30 October 2020 to 30 June 2021. Patients were selected from those who first visited the oral surgery department of Shanghai Ninth Hospital and diagnosed with TMJ ADDwoR and recommended for disc repositioning surgical treatment by the same senior doctor. Inclusion criteria were as follows: (1) patients aged between 10 and 25 years old, including 10 and 25 years; (2) patients with MRI and CT examinations at baseline; (3) patients diagnosed with unilateral or bilateral TMJ ADDwoR based on MRI (Wilke’s Stage III/IV); and (4) patients recommended for disc repositioning surgical treatment via arthroscopic approach. Key exclusion criteria included histories of orthodontic, trauma, or TMJ surgery treatments.

**HIGHLIGHTS**

- This is a controlled, randomized clinical trial focused on the relationship of temporomandibular joints disc position, especially a displaced disc, and condylar bone modeling/remodeling over a long term of 18 months.
- The condylar bone volume increased after an anterior displaced disc reposited (DR) in both adolescents and young adults. Increased bone mainly occurs at the posterior and lateral part of the condyle after DR.
- Anterior displaced temporomandibular joint disc combined with a medial or lateral movement affected the condylar bone resorption pattern.

The sample size calculation was conducted based on the condylar volume change of the joint with or without TMJ arthroscopic discopexy in our previous analysis (data were not published), which average volume change in the non-surgery and surgery group was 0.94 and 166.50 mm^3^, respectively, with SD 153.7 and 201.5 mm^3^. With an *α* value of 0.05, 90% power, and assuming a 10% dropout, a final sample size of 60 patients was required. The sample size was estimated by PASS 15.0 software.

### Control and intervention

Patients were randomly allocated to either the DD group or the DR group. Randomization was performed by independent third-party statisticians using an interactive web response system, with allocation concealment maintained through sealed, opaque, sequentially numbered envelopes prepared by an independent researcher.

Patients in the DD group underwent CT and MRI examinations at the 18-month follow-up without receiving any injectable, occlusal, or surgical interventions during the study period. While permitted to use conservative treatments such as hot compresses or analgesic medications for symptomatic relief at home, these patients received no active therapeutic interventions. Notably, all DD group participants subsequently underwent arthroscopic discopexy upon completion of the 18-month observational period. Patients in the DR group received arthroscopic discopexy surgery followed by CT and MRI evaluations at 18 months postoperatively. All surgeries were performed by a single senior surgeon, and all imaging examinations were conducted and interpreted at the same institution to ensure consistency. The arthroscopic discopexy was performed as described in previous work^[15,19]^. Briefly, the arthroscopic discopexy procedure consists of three primary steps: anterior disc release, reduction, and suturing. For the anterior release, a puncture is made on the anterior surface of the eminence, through which a coblation probe is inserted. A medial-to-lateral incision is then made approximately 2–3 mm anterior to the anterior band of the disc. To prevent damage to blood vessels and nerves, the depth of the anterior release should not exceed 2 mm. Following this, an obturator is used to facilitate disc reduction. Under arthroscopic visualization, a 12-gauge suturing needle is inserted into the border of the bilaminar zone and the posterior band for disc suturing. The needle is passed through the retrodiscal tissue in a medial-to-lateral direction. Simultaneously, a second puncture is made at the anterior wall of the external auditory canal. Customized suture grippers are then introduced through these two punctures to secure the surgical sutures. The sutures are tied with knots placed beneath the cartilage of the external auditory canal, and the skin incision is closed.

### Data collection and outcomes

**Figure 1.**
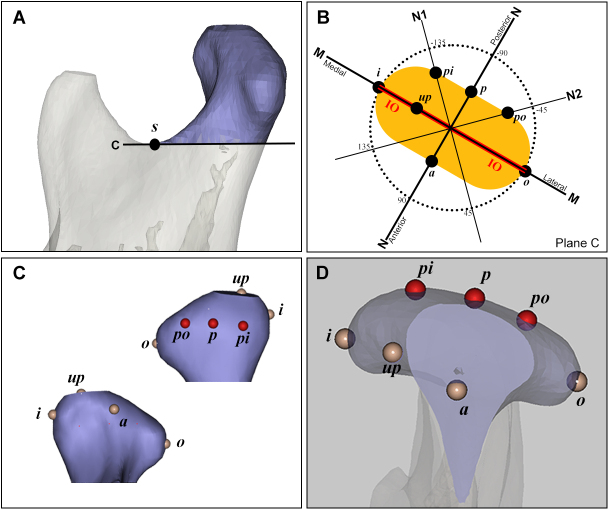
**The method to define 7 points on condylar surface. A.** Condylar bone block was separated from mandibular by plane C; *s*, the lowest point of mandibular sigmoid notch; Plane C, a plane parallel to Frankfort Plane (FH) through point *s*. **B.** The schematic diagram of 7 points’ position: *i*, the innermost point of condylar crest; *o*, the outermost point of condylar crest; M, a plane perpendicular to plane C through points *i* and *o*; IO, a line segment on the plane C, the vertical projection of the line connecting point *i* and point *o*; and N, a plane perpendicular to the IO and plane C through the midpoint of the line segment IO. N1 and N2, two planes perpendicular to plane C and at 45 and −45 degree angles, respectively, to plane N through the midpoint of the line segment IO; *up*, the highest point of the upper edge of the condyle on plane M; *p*, the point where plane N intersects with the lowest edge of the posterior slope of condyle; *a*, the point where plane N intersects with the lowest edge of the anterior slope of condyle; *pi*, the point where plane N1 intersects the lowest edge of the posterior slope of the condyle, closer to point *i*; and *po*, the point where plane N2 intersects the lowest edge of the posterior slope of the condyle, closer to point *o*. **C.** The front and back view of condyle to show the points’ position on the surface. **D.** The vertical view of condyle to show the points’ position on the surface.

The primary outcome was the longitudinal change in the condylar volume from baseline to 18-month follow-up, as quantified through three-dimensional CT reconstructions. Secondary outcomes were topographic changes at seven anatomical landmarks on the condylar surface, measured as vertical height variations from the baseline. All volumetric and linear measurements were derived from CT images. CT images of baseline and 18-month follow-up were imported into the software Mimics 21.0 (Materialise, Belgium). The three-dimensional model of the mandibule was reconstructed first. The condylar bone block was then separated from mandible by an osteotomy plane C, which is parallel to Frankfort Plane (FH) through point *s*, the lowest point of mandibular sigmoid notch, in the software as shown in Figure 1A[20]. The volume of the condylar bone block was calculated[21]. Seven points – point *i*, point *o*, point *a*, point *p*, point *pi*, point *po*, and point *up* – were defined on the condylar surface (Fig. 1B–D). The innermost point of the condylar crest was marked as point *i* and the outermost point as point *o*. Make a plane M perpendicular to the osteotomy plane C through points *i* and *o*. The vertical projection of the line connecting point *i* and point *o* on the osteotomy plane C was the line segment IO. Make a plane N perpendicular to the line segment IO and the osteotomy plane C through the midpoint of the line segment IO. Make two planes perpendicular to the osteotomy plane C and at 45 and −45 degree angles to plane N through the midpoint of the line segment IO, which were marked as plane N1 and plane N2, respectively. The highest point of the upper edge of the condyle on plane M was marked as point *up*. The point where plane N intersects with the lowest edge of the posterior slope of the condyle was marked as point *p*. The point where plane N intersects with the lowest edge of the anterior slope of the condyle was marked as point *a*. The two points where planes N1 and N2 intersect with the lowest edge of the posterior slope of the condyle, the point closer to point *i* was recorded as point *pi*, and the point closer to point *o* was recorded as point *po*. The vertical distance from each point to the plane C was measured and recorded as its height. Mandibular deformity and asymmetry were also assessed based on X-rays. Three indicators were measured as described in the previous study^[8,13]^. The angle of subspinale–nasion–supramentale (ANB), the distance from pogonion to the vertical line passing through Nasion and perpendicular to the FH plane (PN), and the distance from menton to midline (ME). The facial midline was defined as a line perpendicular to the line connecting the left and right intersections of the zygomatico-frontal suture and lateral orbital margin through the crista galli.

### Statistical analysis

All the statistical analyses were performed by SPSS 25.0. The outcome assessors and statisticians were blinded to the group assignment. There were two assessors to do the measurements, and their interobserver concordance (ICC) analysis was performed by SPSS 25.0, and the ICC value was 0.92 with a 95% confidence interval from 0.85 to 0.96 (*P* value < 0.001). For primary and secondary outcomes, the 25% percentile, median, 75% percentile, and mean and standard deviation values were calculated. *Mann–Whitney U* and *t*-tests were used to analyze the difference between groups. All statistical tests were two-sided, and statistical significance was set at *P* value <0.05. Considering the bilateral coupled joints mechanism of TMJ, values of right and left condylar measurements per patient were summed and then analyzed. The general linear model was used to perform the multi-variable analysis.

## Results

### Basic information of patients

**Figure 2.**
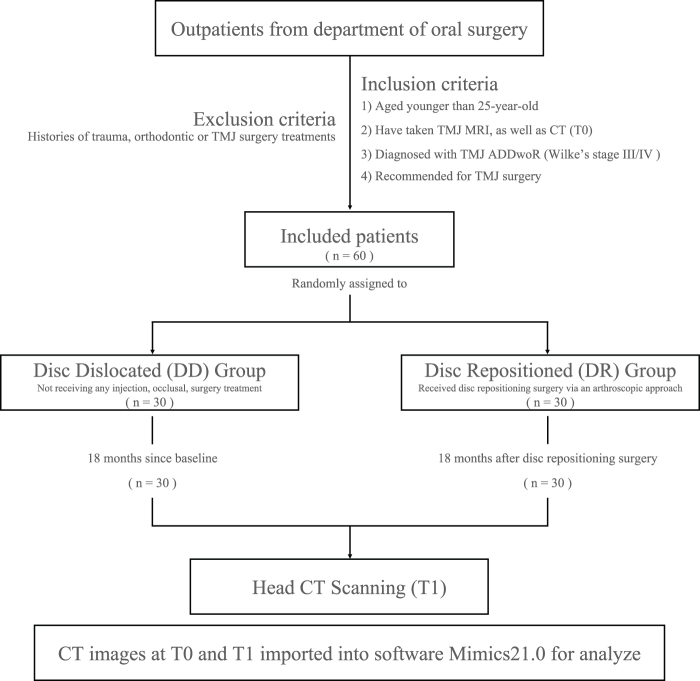
**Trial profile.** T0, time at baseline; T1, time after 18 months.

A total of 60 patients with ADDwoR were included in this study. All patients who underwent arthroscopic disc repositioning surgery remained free of postoperative complications and showed no evidence of disc displacement recurrence throughout the 18-month follow-up period. The information on their age, gender, and affected joints at baseline (T0) were shown in Table 1. 90% of included patients were female. Most of them were aged younger than 18 years (65%). The proportion of unilateral and bilateral ADDwoR was 57% and 43%, respectively. Additionally, 88.3% of them exhibited condylar resorption at T0, as confirmed by MRI findings (Supplemental Digital Content, Table S1, available at: http://links.lww.com/JS9/E708). The study procedure was shown in Figure 2.

**Table 1:** Basic information of patients

	DD *n* (%)	DR *n* (%)	Total *n* (%)
Number of patients	30 (100.0)	30 (100.0)	60 (100.0)
Gender			
Female	27 (90.0)	27 (90.0)	54 (90.0)
Male	3 (10.0)	3 (10.0)	6 (10.0)
Age (year)			
10–18	20 (66.7)	19 (63.3)	39 (65.0)
19–25	10 (33.3)	11 (36.7)	21 (35.0)
TMJ diagnosis			
Unilateral ADDwoR	17 (56.7)	17 (56.7)	34 (56.7)
Bilateral ADDwoR	13 (43.3)	13 (43.3)	26 (43.3)

ADDwoR, anterior disc displacement of temporomandibular joint without reduction; DD, disc displaced; DR, disc reposited; TMJ, temporomandibular joints.

### The condylar volume significantly increased after TMJ disc repositioned

**Figure 3.**
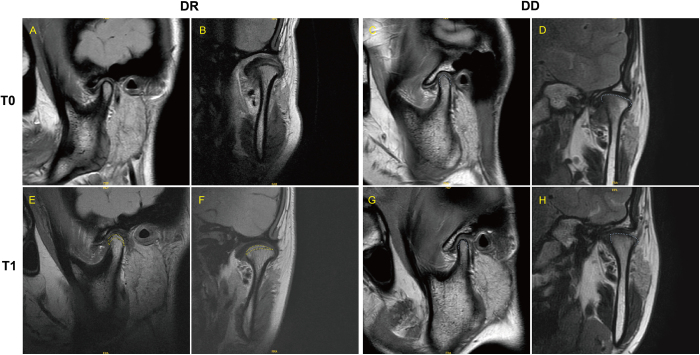
**Morphological change on MRI of condyle in DR and DD groups at T0 and T1. A. C. E. G.** Sagittal view and mouth-closed position of TMJ MRI; **B. D. F. H.** Coronal view of TMJ MRI; yellow dotted line, the new bone formed on condylar surface and blue dotted line, the superior surface border of condyle. DD, disc dislocated group; DR, disc repositioned group; T0, time at baseline; T1, time after 18 months.

At T0, the condylar volume of each patient between DD and DR groups showed no difference, with median summed volume of 2641.1 and 2545.7 mm^3^, respectively (Table 2). At 18-month follow-up (T1), summed condylar volume per patient was significantly increased by 279.6 mm^3^ in the DR group compared with a slight increase of 29.5 mm^3^ in the DD group (*P* < 0.0001). Morphological change of the condyle was shown in Figure 3. To distinguish age groups, patients were categorized into two cohorts: 10–18 and 19–25 years, as shown in Supplemental Digital Content, Table S2, available at: http://links.lww.com/JS9/E709. After 18 months of disc repositioning, the condylar volume increased significantly in both age groups compared to their respective controls. In the DR group, younger patients aged 10–18 years demonstrated an increase of 294.9 mm^3^ (*P* < 0.0001), while older patients aged 19–25 years showed an increase of 270.1 mm^3^ (*P* = 0.0001). In contrast, in the DD group, younger patients aged 10–18 years exhibited a slight increase of 29.5 mm^3^ (*P* < 0.0001), and older patients aged 19–25 years displayed an increase of 16.2 mm^3^ (*P* = 0.0001). The condylar volume and the change of normal and affected joints in unilateral ADDwoR patients were separately analyzed. The condylar volume was significantly decreased in the affected joints (Supplemental Digital Content, Table S3, available at: http://links.lww.com/JS9/E710). The condylar volume of the affected joints increased to 154.47 mm^3^ after DR, compared with its control in the DD group of 35.27 mm^3^ (*P* = 0.0002). On the contrary, the condylar volume change of normal joints showed no difference between DD and DR groups at T1 (Table 3). Multiple linear regression analysis showed a strong positive correlation of reposited disc with condylar volume change value (Table 4). Variables of age, gender, and affected joint(s) did not correlate to condylar bone volume change. Besides, the angle of subspinale–nasion–supramentale (ANB), the distance from pogonion to the vertical line passing through Nasion and perpendicular to the FH plane (PN), and the distance from menton to midline (ME) were measured to evaluate mandibular deformity and asymmetry (Table 5). The distance of ME and PN increased in the DD group and decreased in the DR group, with a *P* value of <0.0001 and 0.0406, respectively. ANB showed no difference between groups.

**Table 2:** Condylar volume at T0 and volume change at T1 (mm^3^)

Group	V_T0_		V_T1-T0_	
	Median	Q25, Q75	Median	Q25, Q75
DD	2641.1	2153.1, 3075.1	29.5	−138.4, 93.8
DR	2545.7	2140.9, 3400.0	279.6	149.5, 434.1
*P* value^a^	0.752		<0.0001****	

DD, disc displaced group; DR, disc reposited group; Q25, quartile 25; Q75, quartile 75; T0, time at baseline; T1, time at 18-month follow-up; V_T0_, the summed volume of left and right condyle per patient; V_T0-T1_, the summed volume change of left and right condyle per patient; –, decreased change in condylar volume.

^a^ *Mann-Whitney U* test, two-tailed.

^****^ *P* value < 0.0001.

**Table 3:** The condylar volume change of patients with unilateral ADDwoR between T0 and T1 (mm^3^)

Joint	DD (*n* = 17)		DR (*n* = 17)		*P* value^a^
	Median	Q25, Q75	Median	Q25, Q75	
Normal	19.92	−116.68, 122.87	111.58	47.32, 220.08	0.0575
ADDwoR	35.27	−102.05, 78.31	154.47	82.72, 267.86	0.0002***
*P* value^a^	0.7596		0.2743		

DD, disc displaced group; DR, disc reposited group; Q25, quartile 25; Q75, quartile 75; T0, time at baseline; T1, time at 18-month follow-up; –, a decreased change.

^a^ *Mann-Whitney* test, two-tailed.

^***^ *P* value < 0.001.

**Table 4:** Multiple linear regression analysis of variables gender, age, disc position, and TMJ diagnosis with summed condylar volume change

Variable	*B*	Standard error	95% CI	*t*	*P* value
Intercept	−4.41	66.37	(−137.42, 128.60)	−0.066	0.947
Gender (Female)	−143.95	105.35	(−355.08, 67.17)	−1.366	0.177
Disc position (Reposited)	354.59	61.33	(231.68, 477.49)	5.782	<0.001
Age (10-18y)	21.93	68.81	(−115.97, 159.82)	0.319	0.751
TMJ diagnosis (Unilateral ADDwoR)	−29.27	64.32	(−158.18, 99.63)	−0.455	0.651

ADDwoR, anterior disc displacement of temporomandibular joint without reduction; TMJ, temporomandibular joints.

**Table 5:** The difference of ME, ANB, and PN of patients between T0 and T1

Indicator		DD		DR		*P* value^a^
		Median	Q25, Q75	Median	Q25, Q75	
ME_T1-T0_	mm	0.975	0.328, 1.920	−0.515	−1.415, 0.108	<0.0001****
ANB_T1-T0_	degree	0.015	−1.078, 1.005	−0.400	−1.883, 0.153	0.1833
PN_T1-T0_	mm	3.945	−1.233, 5.580	−0.145	−4.760, 3.300	0.0406*

ANB, the angle of subspinale-nasion-supramentale; DD, disc displaced group; DR, disc reposited group; ME, the distance from menton to facial midline; PN, the distance from pogonion to the vertical line passing through Nasion and perpendicular to the FH plane; Q25, quartile 25; Q75, quartile 75; T0, time at baseline; T1, time at 18-month follow-up; –, a decreased change.

^a^ *Mann-Whitney* test, two-tailed.

^*^ *P* value < 0.05.

^****^ *P* value < 0.0001.

### Increased bone mainly at posterior and lateral condylar surface after DR

Point *i*, point *o*, point *a*, point *p*, point *pi*, point *po*, and point *up* were 7 points defined on the condylar surface. The height, vertical distance from point to the plane C, of each point was measured. The height of points between two groups at T0 showed no difference (Supplemental Digital Content, Table S4, available at: http://links.lww.com/JS9/E711). After 18 months, the height of all 7 points increased in the DR group (Table 6). In the DD group, 5 of 7 points showed an increase in height and a decrease in height for the medial and lateral point. There were significant increases in the height change of point *o*, point *po*, and point *up* in the patients of DR group compared with those in the DD group. However, the height changes of point *i*, point *a*, point *p*, and point *pi* showed no difference between DD and DR groups. To account for age differences, condylar height and its change were compared separately for patients aged between 10–18 and 19–25 years (Supplemental Digital Content, Table S5, available at: http://links.lww.com/JS9/E712 and Supplemental Digital Content, Table S6, available at: http://links.lww.com/JS9/E713). In both age groups, the increase in condylar height of point *po* was observed, with *P* values of 0.011 and 0.029, respectively. Additionally, younger patients showed increased condylar height of point *o* (*P* = 0.022), while older patients exhibited an increased condylar height of point *pi* (*P* = 0.024). However, when analyzed by age classification, no significant difference was found in the condylar height change of point *up*.

**Table 6:** The height changes of point *i, o, p, pi, po, up*, and between T0 and T1 (mm)

Point	DD		DR		*P* value^a^
	Median	Q25, Q75	Median	Q25, Q75	
D_T1-T0_-*i*	−0.040	−1.085, 1.683	1.020	−0.127,2.043	0.089
D_T1-T0_-*o*	−1.000	−1.955, 0.720	0.425	−0.997, 1.793	0.018*
D_T1-T0_-*p*	0.560	−0.102, 1.440	1.230	−0.272, 2.655	0.389
D_T1-T0_-*pi*	0.705	−0.235, 1.830	1.595	0.237, 3.405	0.077
D_T1-T0_-*po*	0.370	−0.975, 0.780	1.330	0.670, 2.640	0.001^b^
D_T1-T0_-*up*	0.175	−0.437, 0.657	0.940	−0.222, 2.090	0.032*
D_T1-T0_-*a*	0.145	−0.737, 0.730	0.870	−0.370, 2.920	0.122

DD, disc displaced group; DR, disc reposited group; D_T1-T0_, the summed height changes of each mark point on left and right condyle per patient between T0 and T1; Q25, quartile 25; Q75, quartile 75; T0, time at baseline; T1, time at 18-month follow-up; –, a decreased height change.

^a^ *Mann-Whitney* test, two-tailed.

^*^ *P* value < 0.05.

^b^ *P* value < 0.01.

### Anterior displaced TMJ disc combined with a medial or lateral movement affected condylar bone resorption pattern

47.7% of anterior displaced discs accompanied a medial and lateral movement were found based on coronal MRI views (Supplemental Digital Content, Table S7, available at: http://links.lww.com/JS9/E714). The condylar volume and height difference values of point *i*-point *o* and point *pi*-point *po* of each condyle at T0 were calculated. Their condylar volume showed no difference (Supplemental Digital Content, Table S8, available at: http://links.lww.com/JS9/E715). For each joint with ADDwoR, the height difference value of point *pi*-point *po* showed a difference between two types of displaced discs at T0. A larger decrease in the height difference between posteromedial and posterolateral regions for the joint with an anterolateral displaced disc (Table 7) was observed compared with that of the joint with an anteromedial variant.

**Table 7:** The height difference of point *i, o, pi*, and *po* of condyle in affected joint with an anteromedial and anterolateral disc displacement at baseline (mm)

Disc position	Joints	D*_i-o_*	D*_pi-po_*
Anteromedial	23	2.753 ± 1.713	−0.151 ± 1.197
Anterolateral	18	1.719 ± 2.589	−1.068 ± 1.333
*P* value^a^		0.132	0.003**

D*_i-o_*, the height difference between point *i* and point *o* per condyle; D*_pi-po_*, the height difference between point *pi* and point *po* per condyle; –, negative value.

^a^ Unpaired *t* test, two-tailed.

^**^ *P* value < 0.01.

## Discussions

To our knowledge, this is the first randomized controlled trial investigating the long-term relationship between TMJ disc position – particularly a displaced disc position – and condylar bone modeling/remodeling. While previous clinical trials have examined condylar bone remodeling in ADD patients, significant limitations remain. Shen et al. compared occlusal treatment versus combined surgical-occlusal therapy in adolescent ADD patients for 14 months[14]. However, their study lacked a control group without therapeutic intervention and relied solely on MRI-based condylar height measurements, which provide limited assessment of bone remodeling. This study addressed these methodological gaps through several key innovations: (1) inclusion of a DD control group receiving no occlusal, injection, orthodontic, or surgical interventions; (2) comprehensive evaluation of condylar remodeling using both volumetric and linear measurements derived from CT imaging; and (3) extended 18-month follow-up period to better characterize long-term morphological changes associated with different disc positions. This approach provides unprecedented three-dimensional quantification of disc position-related condylar adaptations.

This study revealed significant differences in condylar volumetric change between DD and DR groups in the 18-month observation period. The DR group demonstrated substantial condylar growth, with a median total volume increase of 279.6 mm^3^ per patient at 18 months post-surgery. While the DD group showed a slight median volume increase of 29.5 mm^3^, this finding contrasts with the previous reports of volume reduction in similar populations[13]. Both DD and DR groups showed a tendency of increased condylar height. The observed volumetric increases in the DD group may reflect the remarkable compensatory capacity of condylar bone modeling in adolescents and young adults, potentially overcoming the degenerative effects of persistent disc displacement during extended follow-up periods. This compensatory mechanism could account for the discrepancy between our findings and earlier studies reporting condylar volume reduction. Stratified analyses by age (10–18 years versus 20–25 years) were conducted in this study. The outcomes of unilateral ADDwoR cases were separately analyzed. Following 18 months of disc repositioning, both age groups demonstrated significant condylar volume increase compared to their respective controls. Similarly, affected joints in unilateral cases showed greater condylar volume than their contralateral counterparts. Although pubertal growth velocity may influence condylar height, our longitudinal assessment revealed no obvious inter-age difference in volumetric changes. This null finding may be due to the limited sample size of this study or the bone growth ability into early adulthood[22], as supported by Liu et al.’s demonstration of ongoing condylar bone modeling potential through age 25 years. Mandibular deformity and asymmetry were quantified using ME and PN measurements. The distance of ME and PN increased in the DD group while it decreased in the DR group. These findings collectively indicate that persistent anterior displaced disc constrains condylar growth potential in young patients and disc reposition enables resumption of physiological bone modeling. Also, the adolescent-to-young adult transition maintains significant condylar adaptive capacity.

An innovative CT-based approach was implemented for condylar height measurement in this study, offering distinct advantages over conventional MRI methods. While MRI-based height assessment has been widely utilized[23], its reliance on multiple two-dimensional slices often compromises the accuracy of regional condylar surface measurements. CT images enabled precise three-dimensional condylar reconstruction, permitting exact height measurements at anatomical landmarks. As detailed in the Methods section, 7 anatomically defined points across the condylar surface represent superior, anterior, posterior, medial, lateral, posteromedial, and posterolateral zones to comprehensively evaluate topographic changes. The height difference at each point quantitatively reflected site-specific bone modeling/remodeling patterns. Key findings revealed that the condylar bone modeling/remodeling was different at superior, posterior, and lateral condylar surface between DD and DR groups. Especially, the height of the lateral condylar surface decreased in the DD group compared with the DR group, which the height was increased. Additionally, approximately half of ADD accompanied a medial or lateral disc movement on coronal MRI views. This observation prompted an investigation into whether ADD direction influences condylar bone modeling patterns. Comparative analysis of baseline condylar topography revealed that condyles with anterolateral disc displacement exhibited significantly greater height differential between posteromedial and posterolateral points compared to anterolateral disc displacement cases. This height discrepancy suggests more pronounced bone resorption at the posteromedial condylar surface in anterolateral displacement variants. This finding indicates that the spatial distribution of condylar bone modeling consistently correlated with disc displacement directions. Procedures in reposition surgery for ADDwoR joints may require directional modifications for medial or lateral displacement variants.

Several limitations of this study should be acknowledged. First, the sample size was relatively small, particularly for subgroup analyses. Both unilateral and bilateral affected ADDwoR patients were included in this study. The subgroup analysis of condylar volume changes in unilateral cases was limited to only 17 subjects. This restricted sample size may compromise the statistical power and generalizability of findings. Future studies with larger cohorts focusing specifically on unilateral ADDwoR patients are warranted to more thoroughly investigate whether disc position changes influence long-term bone modeling in the contralateral healthy joint. Also, due to the limited sample size, this study could not analyze height change patterns of condyles with anteromedial or anterolateral disc displacement at 18-month follow-up. Second, while previous studies have considered both 12- and 24-month interval as endpoints for long-term follow-up^[24,25]^, the 18-month period in this study may still be insufficient to fully assess outcomes. However, since patients assigned to the control (DD) group received no active treatment, extending follow-up period beyond 18 months was ethically restricted by both the ethical committee and the participants’ legal guardians. Future studies with extended observation periods may provide more comprehensive insights. Third, this study mainly examined condylar bone modeling and remodeling in adolescents and young adults using imaging-based metrics. Future investigations should incorporate clinical and laboratory indicators to provide a more comprehensive understanding of how joint disc position affects joint function and quality of life.

In conclusion, our findings provided solid evidence that disc position directly influences condylar bone modeling and remodeling patterns in adolescents and young adults. ADDwoR was found to restrict condylar bone growth. Furthermore, distinct bone modeling patterns were observed on the condylar surface depending on the directions of ADD, suggesting that surgical repositioning procedures may require specific modifications to optimize postoperative condylar bone remodeling.

## Data Availability

Data sharing is not applicable to this article.

## Research registration unique identifying number (UIN)

Registration number: ChiCTR-INR-17012135 (https://www.chictr.org.cn/showprojEN.html?proj=20725).
